# An Objective Structured Clinical Examination for Medical Student Radiology Clerkships: Reproducibility Study

**DOI:** 10.2196/15444

**Published:** 2020-05-06

**Authors:** Pedro Vinícius Staziaki, Rutuparna Sarangi, Ujas N Parikh, Jeffrey G Brooks, Christina Alexandra LeBedis, Kitt Shaffer

**Affiliations:** 1 Department of Radiology Boston Medical Center Boston University School of Medicine Boston, MA United States; 2 Department of Radiology Milford Reginal Medical Center Milford, MA United States

**Keywords:** radiology, education, education methods, medical education, undergraduate

## Abstract

**Background:**

Objective structured clinical examinations (OSCEs) are a useful method to evaluate medical students’ performance in the clerkship years. OSCEs are designed to assess skills and knowledge in a standardized clinical setting and through use of a preset standard grading sheet, so that clinical knowledge can be evaluated at a high level and in a reproducible way.

**Objective:**

This study aimed to present our OSCE assessment tool designed specifically for radiology clerkship medical students, which we called the objective structured radiology examination (OSRE), with the intent to advance the assessment of clerkship medical students by providing an objective, structured, reproducible, and low-cost method to evaluate medical students’ radiology knowledge and the reproducibility of this assessment tool.

**Methods:**

We designed 9 different OSRE cases for radiology clerkship classes with participating third- and fourth-year medical students. Each examination comprises 1 to 3 images, a clinical scenario, and structured questions, along with a standardized scoring sheet that allows for an objective and low-cost assessment. Each medical student completed 3 of 9 random examination cases during their rotation. To evaluate for reproducibility of our scoring sheet assessment tool, we used 5 examiners to grade the same students. Reproducibility for each case and consistency for each grader were assessed with a two-way mixed effects intraclass correlation coefficient (ICC). An ICC below 0.4 was deemed poor to fair, an ICC of 0.41 to 0.60 was moderate, an ICC of 0.6 to 0.8 was substantial, and an ICC greater than 0.8 was almost perfect. We also assessed the correlation of scores and the students’ clinical experience with a linear regression model and compared mean grades between third- and fourth-year students.

**Results:**

A total of 181 students (156 third- and 25 fourth-year students) were included in the study for a full academic year. Moreover, 6 of 9 cases demonstrated average ICCs more than 0.6 (substantial correlation), and the average ICCs ranged from 0.36 to 0.80 (*P*<.001 for all the cases). The average ICC for each grader was more than 0.60 (substantial correlation). The average grade among the third-year students was 11.9 (SD 4.9), compared with 12.8 (SD 5) among the fourth-year students (*P*=.005). There was no correlation between clinical experience and OSRE grade (−0.02; *P*=.48), adjusting for the medical school year.

**Conclusions:**

Our OSRE is a reproducible assessment tool with most of our OSRE cases showing substantial correlation, except for 3 cases. No expertise in radiology is needed to grade these examinations using our scoring sheet. There was no correlation between scores and the clinical experience of the medical students tested.

## Introduction

### Background

At our institution, there are approximately 160 to 180 medical students per graduating class, with 15 to 20 students in each 4-week radiology clerkship block, comprising predominantly third- and a few fourth-year medical students. Students receive 1 to 2 hours of daily didactic-style teaching directed toward a weekly rapid-fire quiz on topics including chest imaging, abdominal imaging, musculoskeletal imaging, pediatric radiology, neuroradiology, and nuclear medicine. Throughout the rotation, the medical students also observe residents and faculty in various reading rooms: general radiology, neuroradiology, body imaging, musculoskeletal imaging, pediatric radiology, breast imaging, and interventional radiology.

A variety of methods are used to assess medical students’ performance during clerkships at different institutions. As a result, the final performance evaluation is often a combination of subjective and objective grading techniques. The subjective evaluation involves direct observation of the student performing duties and written assessments or presentations, whereas the objective evaluations include multiple-choice questions such as in Radiology ExamWeb examinations [1] and patient logs. Multiple-choice examinations are the most commonly used, albeit with an only limited assessment of a higher level of knowledge, which would require more complex questions [2,3], while also placing heavy emphasis on recognition and recall. Other limitations often found with multiple-choice examinations include the lack of feedback that test takers receive as well as poor validity [4]. In contradistinction, oral examinations may allow for assessment of a higher level of knowledge and reason but are limited by inconsistency in grading and potential bias [5].

The objective structured clinical examination (OSCE) has been proposed initially by Harden in 1975 as a standard for evaluating medical students’ performance in the clerkship years [6]. The OSCE is intended to evaluate skills and knowledge in a standard clinical setting, and via a preset standard grading sheet, so that clinical knowledge can be evaluated at a high level and in a reproducible way. In a study by Morag et al [7], students’ scores on an OSCE test were shown to increase with additional clinical knowledge. For that reason, many fields of medicine have since demonstrated the OSCE as a useful method to evaluate both medical students and residents [8-11], including radiology [7,12]. In the radiology setting, medical imaging requesting and ordering, imaging interpretation, and the next step in management can be tested for and graded in a single examination.

### Objective

We proposed an OSCE assessment tool designed as an assessment tool for radiology clerkship students. Given the imaging-centered aspect of radiology clerkship, we called it objective structured radiology examination (OSRE). The goal of our proposed tool was to evaluate skills and knowledge in a structured manner, with reproducible results across different examples and different graders. This resource will advance the assessment of radiology clerkship medical students by providing an objective, structured, reproducible, and low-cost method to evaluate radiology clinical knowledge in an OSCE-like format.

## Methods

### Objective Structured Radiology Examination Design

We developed 9 radiology OSRE cases, each with a set of 5 questions for assessment. Initially, for 3 months, we gave these OSRE cases to medical students for preliminary testing. We then openly reviewed the student scorings and reformed the grading sheets to include as many correct and incorrect scorings as possible. For each OSRE case, we designed a scoring sheet with a set of checkboxes corresponding to correct and incorrect scorings. We assigned a point value to each correct or incorrect scoring.

Each OSRE case comprises 1 to 3 radiology images that covered basic radiology diagnoses, followed by a question sheet containing a detailed clinical history and 5 examination questions to be answered in the same sheet. We developed the 5 questions to simulate activities that nonradiology clinicians might perform in a structured fashion: selection of pertinent clinical history needed for filling out imaging requisitions, recognition of clinically important findings, formulation of an overall impression, as well as questions about recommendations and follow-up. We displayed images associated with each OSRE on a projector. The supervisor in the examination room, most commonly a radiology resident, ensured that the image was visible to all. All the case images consisted of radiographs except for a head computed tomography image.

### Objective Structured Radiology Examination Cases

Case 1 included a posterior-anterior (PA) and lateral chest radiograph showing right upper lobe pneumonia. Case 2 included an upright and supine radiograph of the abdomen showing a small bowel obstruction. Case 3 included 3 axial noncontrast computed tomography images at different levels of the brain through a subdural hemorrhage. Case 4 included frontal and lateral radiographs of the wrist showing a distal radial fracture. Case 5 included a portable frontal chest radiograph showing a right pleural effusion. Case 6 included a supine radiograph of the abdomen showing a feeding tube in the right lower lobe bronchus. Case 7 included a single cross-table radiograph of the knee with a fat fluid level in a large suprapatellar effusion. Case 8 included a PA and lateral radiograph of the chest showing right lower lobe pneumonia. Finally, case 9 included an upright and supine radiograph of the abdomen, showing a small bowel obstruction. All these cases had been previously published at *MedEdPORTAL* as free downloadable resources [13]. Multimedia Appendix 1 is a template for an OSRE case.

### Objective Structured Radiology Examination Grading

The OSRE scoring sheets comprised checklists with specific point values for correct and incorrect scorings. Each question’s score was worth between 1 and 4 points. Many of the individual questions allowed for multiple scorings. For example, 1 question in an OSRE asked students to describe the pertinent positive and negative findings on the chest radiograph displayed on the projector. Students were given positive points for defined correct scorings and negative points for defined incorrect scorings. The highest possible score on the OSRE tests ranged from 24 to 26 points. However, we established that the lowest score possible for any individual OSRE case was 0 even when the number of points amassed was negative. Multimedia Appendix 2 is a template for a scoring sheet.

### Study Design

We obtained institutional review board approval to conduct educational research using students enrolled in the radiology clerkship during an entire academic year, and the need to acquire consent from each medical student was waived. Our study was designed and performed following the Declaration of Helsinki. At the beginning of each block, we informed the students of the research project and told them that their scores from the OSRE cases would not count toward their final grade. The students in each block were taught the standard curriculum throughout their radiology rotation without any specific teaching toward the newly designed OSRE.

There were 11 four-week clerkship blocks (ie, classes) of students in total during the entire year, representing 11 months across the year. At the end of each of the initial 3 weeks of their 4-week block, all the students from the same class were given 1 OSRE. Therefore, each student completed a total of 3 OSRE cases during their rotation at the end of each of the first 3 weeks of the course. The exception was block 11, when these students had only 2 OSREs, instead of 3. All 9 OSREs were given in order. Blocks 1, 4, 7, and 10 took cases 1, 2, and 3; blocks 2, 5, and 8 took cases 4, 5, and 6; and blocks 3, 6, and 9 took cases 7, 8, and 9. Again, as an exception, block 11 was given only cases 4 and 6. We chose this design to spread out the 9 different OSCEs across the entire year in a uniform fashion.

Five different examiners graded each of the OSREs independently for every single medical student. Grader 1 and grader 3 had 1 to 2 years of experience in medical student education. Grader 2 had over 20 years of medical student education experience. Grader 4 was a second-year radiology resident, and grader 5 was a medical student. These graders were selected with the aim of sampling graders at various stages of medical education.

We graded a subset of the OSREs (3 random sets of OSRE tests) a second time, approximately 2 months after completion of the academic year, to assess internal consistency between the graders and reproducibility of our assessment tool.

There was no specific training or instruction for graders, as we designed the test and grading to be self-explanatory based on the scoring sheets. Each examination took approximately 30 seconds to 1 min to grade. We gave students their scores and individualized formative feedback on their OSRE performance at the midclerkship review and final course feedback session as part of the standard process at the radiology clerkship at our institution. Any questions the medical students had regarding the OSRE questions and scorings were answered.

### Statistical Analysis

The reproducibility of our OSRE was assessed by performing interrater reliability with a two-way mixed effects intraclass correlation coefficient (ICC) to determine consistency between the 5 graders. Reproducibility for each grader was also evaluated with an ICC test 2 months later. Utilizing the classification system for ICCs by Landis and Koch [14], an ICC below 0.4 was classified as poor to fair, an ICC of 0.41 to 0.60 was considered moderate, an ICC of 0.6 to 0.8 was substantial, and an ICC greater than 0.8 was almost perfect. An ICC of 0.6 or more was considered a significant correlation.

We also sought to find if there was an association with a higher OSRE score and clinical experience with block number and with the medical school year. For this, we used a multivariate linear regression model in which the mean OSRE score was the outcome variable and the year block was the explanatory variable, with the medical student year as a controlling covariate. We then compared mean OSRE grades between the third- and fourth-year medical students using a two-sided Student *t* test.

## Results

### Summary of Scores

A total of 181 medical students were included in this study, 156 third-year medical students and 25 fourth-year medical students. OSRE score averages by blocks and cases are depicted in Tables 1 and 2, respectively.

**Table 1:** Mean objective structured radiology examination scores for blocks.

Block	Score, mean (SD)
1	13.0 (4.1)
2	8.3 (3.60)
3	12 9 (6.2)
4	15.1 (3.6)
5	11.5 (4.3)
6	12.6 (5.1)
7	13.3 (4.2)
8	9.2 (3.8)
9	12.2 (4.9)
10	14 (4.2)
11	9 (4.1)

**Table 2:** Mean objective structured radiology examination scores for cases.

Case	Score, mean (SD)
1	15.1 (4.1)
2	12.8 (4.5)
3	13.6 (3.5)
4	8.6 (4.0)
5	8.5 (3.2)
6	11.4 (4.3)
7	6.4 (3.2)
8	15.9 (4.0)
9	14.5 (3.0)

### Reproducibility

Interrater reliability was shown to be ranging from poor to substantial average ICCs, with an average range of 0.36 to 0.80 (*P*<.001; Table 3). In most cases, 6 out of 9 showed correlation values of at least 0.6 (substantial correlation). However, case 3 had a poor correlation, and cases 6 and 7 showed moderate correlation.

Grader consistency on the 3 random OSRE cases (cases 1, 2, and 3) after 2 months showed that 4 out of the 5 graders had an ICC equal to or greater than 0.8 (substantial correlation), whereas grader 3 had an ICC of 0.68. Comparing these regraded exams, the range of mean raw score differences was −1 to 0.8 (Table 4). These data illustrate the reproducibility of the grading.

**Table 3:** Interrater reliability among graders with intraclass correlation coefficients.

OSRE^a^		Correlation	95% CI	*P* value
**OSRE 1**				
	Block 1	0.80	0.64 to 0.92	<.001
	Block 4	0.71	0.52 to 0.87	<.001
	Block 7	0.72	0.53 to 0.88	<.001
	Block 10	0.65	0.41 to 0.86	<.001
	Average	0.72	N/A^b^	N/A
**OSRE 2**				
	Block 1	0.79	0.64 to 0.9	<.001
	Block 4	0.79	0.63 to 0.91	<.001
	Block 7	0.90	0.81 to 0.96	<.001
	Block 10	0.73	0.54 to 0.88	<.001
	Average	0.80	N/A	N/A
**OSRE 3**				
	Block 1	0.28	0.08 to 0.56	<.001
	Block 4	0.31	0.1 to 0.6	<.001
	Block 7	0.53	0.31 to 0.76	<.001
	Block 10	0.32	0.11 to 0.61	<.001
	Average	0.36	N/A	N/A
**OSRE 4**				
	Block 2	0.76	0.58 to 0.89	<.001
	Block 5	0.81	0.66 to 0.92	<.001
	Block 8	0.80	0.62 to 0.92	<.001
	Block 11	0.67	0.46 to 0.85	<.001
	Average	0.76	N/A	N/A
**OSRE 5**				
	Block 2	0.76	0.58 to 0.89	<.001
	Block 5	0.54	0.32 to 0.76	<.001
	Block 8	0.53	0.29 to 0.78	<.001
	Average	0.61	N/A	N/A
**OSRE 6**				
	Block 2	0.50	0.27 to 0.74	<.001
	Block 5	0.37	0.16 to 0.64	<.001
	Block 8	0.51	0.26 to 0.77	<.001
	Block 11	0.50	0.27 to 0.74	<.001
	Average	0.47	N/A	N/A
**OSRE 7**				
	Block 3	0.32	0.12 to 0.59	<.001
	Block 6	0.56	0.35 to 0.77	<.001
	Block 9	0.64	0.45 to 0.81	<.001
	Average	0.50	N/A	N/A
**OSRE 8**				
	Block 3	0.61	0.4 to 0.8	<.001
	Block 6	0.54	0.34 to 0.75	<.001
	Block 9	0.76	0.61 to 0.88	<.001
	Average	0.64	N/A	N/A
**OSRE 9**				
	Block 3	0.77	0.6 to 0.9	<.001
	Block 6	0.61	0.41 to 0.8	<.001
	Block 9	0.77	0.61 to 0.9	<.001
	Average	0.72	N/A	N/A

^a^OSRE: objective structured radiology examination.

^b^N/A: not applicable.

**Table 4:** Reproducibility by grader consistency after 2 months.

Grader		Correlation	Difference	*P* value^a^
**Grader 1**				
	OSRE^b^ 1	0.88	0.50	.30
	OSRE 2	0.92	0.39	.35
	OSRE 3	0.85	0.50	.19
	Average	0.88	0.46	N/A^c^
**Grader 2**				
	OSRE 1	0.89	−1.34	*.02*
	OSRE 2	0.85	−2.06	*<.001*
	OSRE 3	0.73	0.25	.70
	Average	0.82	−1.05	N/A
**Grader 3**				
	OSRE 1	0.35	−0.84	.43
	OSRE 2	0.89	1.06	*.03*
	OSRE 3	0.79	−0.69	.11
	Average	0.68	−0.16	N/A
**Grader 4**				
	OSRE 1	0.95	0.06	.83
	OSRE 2	0.82	0.50	.37
	OSRE 3	0.78	−0.44	.39
	Average	0.85	0.04	N/A
**Grader 5**				
	OSRE 1	0.86	0.60	.24
	OSRE 2	0.88	1.06	*.03*
	OSRE 3	0.66	0.69	.25
	Average	0.80	0.78	N/A

^a^Italicized values were statistically significant.

^b^OSRE: objective structured radiology examination.

^c^N/A: not applicable.

### Scores and Clinical Experience

The average OSRE score among all students was 12 (SD 4.9). The average grade among third-year students was 11.9 (SD 4.9), compared with 12.8 (SD 5) among fourth-year students (*P*=.005). There was no correlation between the block number and OSRE score. On the multiple linear regression, the block had an effect of −0.02 (95% CI −0.08 to 0.04; *P*=.48), adjusting for the medical school year (Table 5).

**Table 5:** Multiple linear regression showing the association of block with the objective structured radiology examination score, adjusting for the medical school year.

Variables	Estimate (95% CI)	*P* value^a^
Intercept	9.3 (7.4 to 11.2)	*<.001*
Block	−0.02 (−0.08 to 0.04)	.48
Year	0.9 (0.3 to 1.5)	*.01*

^a^Italicized values were statistically significant.

## Discussion

### Presentation of the Material

In summary, our OSRE assessment resource comprises a set of 9 cases that include 1 to 3 images each, a clinical scenario, and structured questions, along with a standardized scoring sheet that allows for an objective, structured, and low-cost assessment of radiology clerkship medical students. The structured questions aim to assess medical students’ ability to understand history and indication, to describe imaging findings, to give an imaging impression or diagnosis, and to come up with the next step in management.

We found that our OSREs achieve their goal of being objective, structured, reproducible, and low cost. Most cases demonstrated a substantial interrater correlation (6 out of 10 showing an ICC of 0.6 or more). However, the correlation varied from poor to substantial, ranging from 0.36 to 0.80. The graders provided reproducible scores, even after 2 months, with a substantial interrater correlation (above 0.6). Finally, we did not find a correlation between the OSRE scores and clerkship block, but we did see that fourth-year medical students scored better than third-year medical students.

### What We Observed and Lessons Learned

In assessing the different OSRE cases, we found that OSRE cases 3, 6, and 7 had a poor-to-moderate correlation. As all the remaining OSRE cases had an ICC value of more than or equal to 0.6, we still feel that our OSRE is a reproducible testing resource. All graders were consistent, shown by the very small variability in average scores (−1 to 0.8) when the graders regraded the same subset of 3 cases 2 months apart. Our use of various graders with differing medical education backgrounds demonstrates that expertise in radiology is not necessary to grade these examinations. If a consistent and clear grading sheet is used, grading can be performed by anyone with knowledge of medical terminology.

Regarding the association of grade and clinical experience, scores are not supposed to improve with later blocks, as this would mean that they either depend on the overall clinical experience or that students could be sharing the cases or questions with future students, thereby giving them a leg up by providing examination information to their colleagues. We found that scores did not vary with the block, adjusting for the medical school year. However, the fourth-year students had a slightly better average grade than the third-year students, which makes intuitive sense.

Other studies have found the OSCE to provide valuable feedback as well [4,7,15]. In a study of 122 medical students by Morag et al [7], the authors concluded that the OSCE cases provided an opportunity for feedback, by uncovering deficits in individuals. Students were able to review their performance in different clinical topics (chest pain, abdominal pain, etc) as well as types of questions (selection of imaging modality and anatomy) with ease. An unforeseen benefit of our OSRE implementation was that having the OSRE results weekly allowed the clerkship director and assistant to carefully examine areas where students displayed deficiencies or gaps in knowledge as well as to give each student more information on areas of strength and weakness at both the midcourse feedback session and the final course feedback session.

Agarwal et al [15] point out that radiology should incorporate OSCEs as a part of its examination and explain how an ideal radiology OSCE could look like. Specifically, they describe an OSCE method with 10 to 20 stations, some manned and other unmanned, each evaluating activities related to specific radiology topics, for instance, a basic task such as loading a radiograph (radiography OSCE station) or demonstrating an examination technique, such as performing an ultrasound examination of the abdomen in a patient. Completion of a 5-min task within a single station would involve either demonstrating a task to an examiner, providing verbal answers, or writing specific objective answers in a response sheet [15]. Their approach is different than the one we propose here, as our OSRE is a much simpler proposal, albeit less expensive and difficult to implement.

### Limitations

Although this assessment tool has several advantages, it is not devoid of limitations. For instance, our interrater correlation was not substantial for all cases. This could have been remediated with previous training of the graders. However, we opted not to train graders, as training would artificially increase the interrater correlation of the grading process. Although it would be ideal to train graders before they score examinations, graders in real-life settings (such as teachers) may not always get the appropriate training to score the OSCEs.

Another issue was that this evaluation occurred at a single institution. Despite being low cost, the successful implementation of this assessment model requires informatics facilities to hold OSRE documents, including images, cases, and scoring sheets, which need diligent organization. In our institution, we have a clerkship coordinator and 2 volunteering second-year radiology residents to help coordinate the evaluation of medical students. Finally, we should be aware that medical students can use a recall system to convey the OSRE case to the medical students of future blocks. For this reason, there is a need to constantly create new cases.

Our choices of cases and questions are also a limitation. Although multiple modalities were selected, there were no normal cases, and they were a very small selection of the medical students’ radiology clerkship curriculum. In addition, each case had only a few questions, and several other questions could have been included. For example, we could ask students about normal anatomy or imaging pitfalls or even ask them to provide the appropriate history to order an imaging examination. Different OSREs can be created to assess different skills in the radiology specialty such as the use of clinical guideline algorithms (eg, Breast Imaging-Reporting and Data System or what to recommend for an incidental finding), dictation and descriptive skills, differential diagnosis, or next step in management, among others. However, these skills would be most appropriate to radiology residents, not to medical students.

OSCEs are an excellent method to evaluate medical students, but they work best when they aim to evaluate a clinical, especially manual, skill. In contradistinction, the output of a radiologist is usually a report, which can be written in subjective ways. Structured radiology reports and modern template standards are useful to make our reports more objective, but they do not reduce the inherent subjectivity of the radiologist evaluating an imaging examination. This means that the main activity of a radiologist cannot be evaluated with the OSRE described here. On the other hand, the goal of a radiology clerkship is not to train a radiologist. Rather, it aims to teach and evaluate concepts that underlie the foundations of radiology, which should be assessed more objectively whenever possible.

### The Next Steps

Given these limitations, there are many areas of improvement and ways to refine this resource, for instance, by expanding our questions and our cases, as described above. Furthermore, this model can be enhanced by making it all computer based, with a cloud-based storage software on the web. If we create a large online database of hundreds of OSRE cases or more, a piece of software could download a random case for each student. This could lead to the expansion of this model to other institutions. If multiple institutions are interested in this endeavor, it could remain to be a low-cost model.

Finally, future studies are needed to assess the validity of this tool compared with the standard means of assessing knowledge, including multiple-choice questions. Additional research could also assess the validity of OSRE-style examinations in radiology clerkships with a larger number of institutions and medical students.

## Appendix

Multimedia Appendix 1 Template for a case.

Multimedia Appendix 2 Template for a scoring sheet.

## Abbreviations

ICC: intraclass correlation coefficient
OSCE: objective structured clinical examination
OSRE: objective structured radiology examination
PA: posterior-anterior
