# Supplementary material for: An Objective Structured Clinical Examination for Medical Student Radiology Clerkships: Reproducibility Study
Source: JMIR Med Educ. 2020 May 6;6(1):e15444. doi: 10.2196/15444 (PMC7240440; doi:10.2196/15444)
Supplement: Multimedia Appendix 1 [file mededu_v6i1e15444_app1.doc]

**EXAMPLE OSCE Student name: ______________**

History paragraph

1. Example: WHAT will you list as the clinical indication?

_____________________________________________________________

2. Example: DESCRIBE the findings including pertinent positives and negatives on the images.

______________________________________________________________

______________________________________________________________

______________________________________________________________

3. Example: WHAT diagnoses would you consider (list as many as seem appropriate)?

Circle the diagnosis you think is most likely

______________________________________________________________

______________________________________________________________

4. Example: WHAT would you do next in terms of imaging (the answer may be “nothing”)?

5. Extra question pertinent to the case.

____________________________________________________________________________
