# Supplementary material for: An Objective Structured Clinical Examination for Medical Student Radiology Clerkships: Reproducibility Study
Source: JMIR Med Educ. 2020 May 6;6(1):e15444. doi: 10.2196/15444 (PMC7240440; doi:10.2196/15444)
Supplement: Multimedia Appendix 2 [file mededu_v6i1e15444_app2.doc]

| OSCE SCORE SHEET: CASE NUMBER- X | | | | | Student: | | | | | |
| --- | --- | --- | --- | --- | --- | --- | --- | --- | --- | --- |
| QUESTION 1- (MAX ) | | | | | | QUESTION 2- (MAX ) | | | | |
| **Pertinent Hx (1)** | | **Extraneous Hx (-1)** | | **0-5** | | **Appropriate Terms (1)** | | **Incorrect Terms (-1)** | | **0-5** |
| Age |  |  |  |  | |  |  |  |  |  |
| Gender |  |  |  |  |  |  |  |  |  |  |
|  |  |  |  |  |  |  |  |  |  |  |
|  |  |  |  |  |  |  |  |  |  |  |
|  |  |  |  |  |  |  |  |  |  |  |
|  |  |  |  |  |  | **Accurate location/ Pertinent negatives (1)** | | **incorrect location (-1)** | | **0-4** |
|  |  |  |  |  |  |  |  |  |  |  |
|  |  |  |  |  |  |  |  |  |  |  |
|  |  |  |  |  |  |  |  |  |  |  |
|  |  |  |  |  |  |  |  |  |  |  |

| QUESTION 3- (MAX ) | | | | | QUESTION 4- (MAX ) | | | | |
| --- | --- | --- | --- | --- | --- | --- | --- | --- | --- |
| **Appropriate Ddx** |  | **Unlikely Ddx (-1)** |  | **0-5** | **Appropriate rec (2 pts each)** | | **Inappropriate rec**  **(-1 pt each)** | | **0-4** |
| **Best answer – (score)** |  |  |  |  |
|  |  |  |  |  |  |  |  |  |
|  |  |  |  |  |  |  |  |
|  |  |  |  |  |  |  |  |
|  |  |  |  |  |  |  |  |
|  |  |  | |  |  |  |  |
| Question 5 – (MAX )   | **Appropriate recommendation** |  | **Inappropriate recommendation** |  | **0-2** | | --- | --- | --- | --- | --- | |  |  |  |  |  |   GRAND TOTAL SCORE | | | | | | | | |  |
